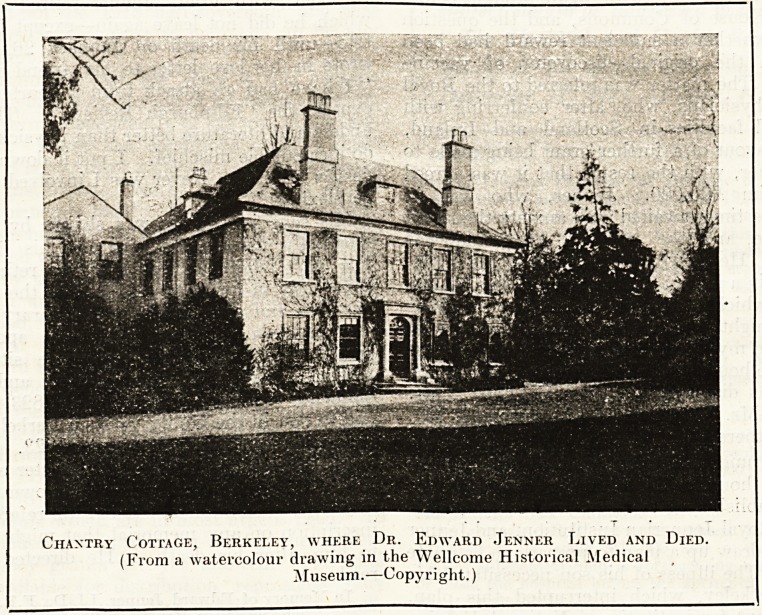# Edward Jenner—II

**Published:** 1921-12

**Authors:** C. J. S. Thompson

**Affiliations:** M.B.E., Curator of the Wellcome Historical Medical Museum


### EDWARD JENNER.

### THE DISCOVERER OF VACCINATION.—II.

By C. J. S. THOMPSON, M.B.E., Curator of the Wellcome Historical Medical Museum.

THE news of Jenner's discovery next spread over the Continent of Europe, and vaccination was demonstrated in Vienna by De Carro in 1799. Towards the end of the year 1799, Jenner's "Inquiry" was translated into German by Ballhorn, who together with Stromeyer introduced the practice of vaccination into Hanover. Valentin and Desoteux were the first to call attention to the subject in France, and in 1800 Liancourt established a vaccine institution by subscription, obtaining considerable financial support from Lucien Bonaparte, who was then Secretary of the Interior. François Colon, a physician of Paris, had his own son vaccinated, a baby of eleven months, in order to encourage those who hesitated. With enthusiasm worthy of the cause, he wrote a pamphlet, which had a wide circulation, in which he offered to inoculate the poor gratuitously, and all soldiers and their children who had not had smallpox, on a simple letter of recommendation from beneficence committees, from different administrations and constituent bodies. It may be interesting here to note that the word "vaccination" was first applied to Jenner's method of cowpox inoculation in France.

EDWARD JENNER.
(From an oil painting in the Wellcome Historical Medical Museum.—Copyright.)

In January 1800, Jenner's original treatise, "An Inquiry into the Cause and Effects of the Variolæ Vaccinæ," was translated into French by the Count de la Roque, and five years later Napoleon Bonaparte demonstrated his confidence in Jenner's theories by issuing an order that all soldiers in his army who had not suffered from smallpox were to be vaccinated. This was the first instance of compulsory vaccination.

Towards the close of 1800 vaccination was introduced into Holland by Dr. Davids, of Rotterdam. He first journeyed to Paris for the purpose of seeing the practice carried out, where he obtained some virus, which he took back to Rotterdam, but his first attempts failed. Subsequently, he received a supply from England through Boulogne, which proved efficient. Davids translated Jenner's pamphlet "An Inquiry" into Dutch, which was published about this time, and soon became known throughout the Netherlands.

The importance of vaccination was soon realised and taken up with enthusiasm also in Switzerland, Italy, and Spain. In the last country its possibilities were recognised, and the Government in 1803 despatched an expedition for the purpose of introducing the practice of vaccination throughout the Spanish possessions of the Old and New Worlds, where smallpox was constantly raging. The vessel in which the expedition sailed carried twenty-three unvaccinated children, who were to be vaccinated on the voyage in order to preserve the lymph active by passing it from arm to arm.

Italy also was not slow to follow Spain in adopting Jenner's discovery, and the practice was successfully exploited by Sacco, of Milan, in 1801. He laboured with unwearied activity, becoming the Director of Vaccination, and in a few years had vaccinated 20,000 of his countrymen. Much of the vaccine used was obtained from an animal that had natural cowpox, which was discovered after a prolonged search in Lombardy.

In Sicily and Naples, where smallpox was rife, vaccination was received with great enthusiasm, religious ceremonies being formed for the purpose of receiving the "blessed vaccine," as it was termed.

In Russia Jenner's discovery was taken up with still greater enthusiasm, and among its most earnest supporters was the Empress Alexandra, who personally urged her subjects to be vaccinated,

**Edward Jenner**—(*cont.*).

and ordered that the first child to submit to the operation should receive the name of "Vaccinoff" and be educated at the public expense. The fortunate child who was thus duly named became a kind of national hero, and, after vaccination, was conveyed to St. Petersburg in one of Her Majesty's Imperial coaches. He was educated in the Foundling Hospital in the capital, and afterwards received a pension for life. The Empress, in commemoration of this, afterwards presented Jenner with a valuable diamond ring. Thus Jenner's influence and popularity grew apace, especially on the Continent of Europe.

On his petition, the Emperor of Austria and the King of Spain released Englishmen who had been taken prisoners in the war. In France, where a Dr. Wickham remained a prisoner, Jenner was applied to by one of his friends to present a petition to Napoleon soliciting the doctor's liberation. He good-naturedly undertook the task, and drew up a petition to the Emperor just at the time when he was exhibiting his greatest animosity towards Britain. The petition was forwarded, and it so happened to be handed to him when he was sitting in his carriage together with the Empress Josephine while waiting for their horses to be changed. Glancing at it, he exclaimed to the coachman, "Away! Away!"; but the Empress, examining the paper, said: "But stay, you see from whom this comes, Jenner." Napoleon's manner is said to have changed immediately, and he replied, "What that man asks is not to be refused," and so Dr. Wickham obtained his release. The success of vaccination in the French Army led Napoleon later on to issue a decree that 100,000 francs should be placed at the disposal of the Minister of the Interior for the propagation of vaccination.

Directly Jenner's discovery was known in India intense anxiety was shown to obtain virus to carry out vaccination in that country, where smallpox was so prevalent, and where its effects had been so deadly, but the difficulty was in forwarding the vaccine such a great distance in those days, so that it could reach its destination in an active condition. Many trials were made of various methods. Threads dipped in the virus were enclosed between glass plates, linen was impregnated, lancets of silver, steel, gold,* and ivory were tried in turn, and, after a series of trials, ivory was found to be the best medium for transporting the vaccine. On ivory lancets, therefore, it was first despatched from Breslau to Moscow, and other prepared lancets of silver, silver-gilt, and ivory, also lint impregnated with vaccine enclosed between pieces of glass, then coated with wax, were conveyed across the Bosphorus, through the Desert to the banks of the Tigris, where they were received on March 31, 1802. Hence they were forwarded by Dr. Short, who received them there, to Mr. Milne, surgeon to the British Consul at Bassora or Basra. On June 17 he vaccinated forty persons with the virus successfully, including the crews of some vessels departing for Bombay. Thus the vaccine was carried across the ocean by man, and before the end of June it reached India.

In the year 1803 the Governments of Sweden and Denmark so effectually enforced the practice of vaccination that smallpox became unknown in those countries, and they remained free from the disease for nearly twenty years.

During the years that Jenner had spent upon his research work on cowpox and vaccination he had expended a considerable amount of time and money, with the result that his own practice had to be neglected. Meanwhile he was hoping that his discovery might eventually recoup him and be made a financial success. His straitened means becoming known to his friends, he was advised to apply to Parliament for a grant, which he decided to do, and on December 9, 1801, he journeyed to London to frame a petition, for which he obtained a promise of assistance from Admiral Berkeley. The petition came before the House of Commons in the March of the following year, and was presented on the following grounds: First, that Jenner had discovered that cowpox was inoculable from cow to man; and, secondly, that persons so inoculated were for life perfectly secure from smallpox. Jenner further claimed that he had not made a secret of his discovery, that the progress of smallpox had already been checked, and that he had been put to much expense and anxiety.

The matter was referred to a Committee, and in June 1802 a report was laid before the House of Commons, which ultimately granted £10,000 to Jenner, and he returned to his country home at Berkeley.

Shortly after this some of Jenner's leading supporters in London endeavoured to form a Jennerian Institution for promoting universal vaccination. The idea was taken up with enthusiasm, and the Queen became the patron, and King George III. granted permission for the Society to be called "The Royal Jennerian Society for the Termination of the Smallpox." An influential Board of Directors, together with a Medical Council, was appointed. Jenner was elected the first President, and Dr. John Walker appointed Resident Vaccinator. Thirteen vaccinating stations were opened in London, and in eighteen months it was announced that 12,288 inoculations had taken place and 19,352 charges of cowpox virus had been supplied to different parts of the British Empire and foreign countries. Although this Institution was most successful at first, yet, mainly owing to friction among the staff, in six years its popularity seemed to wane. In the end Jenner came to a disagreement with the chief vaccinator, who resigned his office, and in 1808 the Society practically collapsed.

Jenner's name being now well known and established, he decided to leave the country and to

* Jenner alludes to the gold-bladed lancets for the transmission of the lymph in a letter written from Berkeley on December 7, 1803, as follows: "Pray request him (Mr. Fewster) to send me on the point of two or three toothpicks or on the gold lancet I enclose, a little of the vaccine matter he is now using, which I believe is from my original stock."

**Edward Jenner—(*cont.*).**

commence practice in London. He took a house in Hertford Street in the West End of London. He soon found out, however, that the fact of being celebrated as a discoverer did not mean that he would make a successful medical practitioner, and he was disappointed in the results. So, after a few years' trial, he gave it up, and returned to his native village. In a letter he wrote to his friend a short time after his residence in London, he says:—

> I have now completely made up my mind with respect to London. I have done with it, and have again commenced the village doctor. I found my purse not equal to the sinking of the £1,000 annually (which has actually been the case for several successive years) nor the gratitude of the public deserving of such a sacrifice. You heard, after what I had done, the toils I have gone through, and the anxieties I have endured in obtaining for the world a greater gift than man has ever bestowed on them before (excuse this burst of egotism) to be thrown by with a bare remuneration of my expenses.

About this time failures of vaccination multiplied considerably, and even some of Jenner's best friends began to lose confidence. His time at Berkeley was largely taken up in replying to correspondence and endeavouring to account for the numerous failures reported. He had always been aware that smallpox could occur after vaccination, but if it did occur he believed that the vaccination could not have been properly performed. He still continued to vaccinate all the poor who applied to him on certain days, so he had sometimes as many as three hundred persons waiting at his door.

Owing to the frequent complaints that reached Jenner of persons practising vaccination who did not implicitly follow his directions, and thus failing through ignorance, on July 1, 1801, he published a statement in which he laid down what he calls the "Golden Rule," which he hoped would tend to make practitioners more careful in their practice.

He declaimed against allowing an unlimited time for taking the vaccine virus from the pustule, maintaining that this ought to be done at an early period of its formation and before the appearance of the areola. He also insisted on the rule that when the pustule was excited, it should be permitted to go through all its stages in an uninterrupted manner. If any deviation appeared in its progress, he always forbade the employment of virus from such a pustule for further inoculations. Jenner's method of vaccinating was based almost exactly on the earlier practice of inoculation, the cowpox matter being inserted under the skin of the arm by a lancet point. Notwithstanding the success and support that his discovery was now receiving in all parts of the world, there were still many prejudiced against it who opposed the practice, and demonstrations, caricatures, and broadsides were published by the anti-vaccinators. Some of the theories put forward against vaccination were of the most absurd description. People actually alleged that those inoculated with cowpox might assume the "bovine features of the animals themselves." Another anti-vaccinist records in a pamphlet the story of a lady who complained that since her daughter was inoculated she "coughed like a cow, and had grown hairy all over her body." Others declared that inoculation with cowpox had been discontinued in some parts of the country because those who had been inoculated in that manner "bellowed like bulls." It was stigmatised

Chantry Cottage, Berkeley, where Dr. Edward Jenner Lived and Died. (From a watercolour drawing in the Wellcome Historical Medical Museum.—Copyright.)

**Edward Jenner—(*cont.*).**

by others as the "damnest thing ever proposed," and the "most degrading relapse of philosophy that ever disgraced the civilised world." Pictures, coloured prints, and pamphlets ridiculing vaccination were published in Great Britain and France, but notwithstanding all opposition the propaganda made steady progress, and soon every country vied with another in honouring its discoverer. Jenner was elected a member of nearly all the leading scientific societies in Europe, and presented with the Freedom of the cities of London, Dublin, Edinburgh, and Glasgow. The Medical Society of London conferred on him a gold medal at their anniversary festival, when Dr. Lettsom, who was then President, delivered an oration on vaccination.

In July 1806 the subject was again brought before the British House of Commons, and the question was raised whether a sufficient reward had been bestowed on the original discoverer of vaccine inoculation. The matter was referred to the Royal College of Physicians, who, after conferring with other medical faculties in Scotland and Ireland, reported in favour of a further grant being made to Edward Jenner, with the result that it was agreed to award him £20,000. Jenner, who was in London at the time, awaited with anxiety the result of the debate, and the relief to his mind was incalculable. He conveyed the news to his sister, Mrs. Black, in a letter: "Pray excuse this shabby bit of paper which I catch up to tell you that Parliament last night voted me the sum of £20,000 for making public my Vaccine Discovery. The debate continued two hours and a-half, during which much eloquence was displayed by Lord H. Petty, Mr. Wilberforce, Mr. Windham, Mr. Whitbread, Mr. Smith, and others."

The Government, having decided to support vaccination, thought the time had now come to found an establishment to revive and carry on the work of the Royal Jennerian Institution, and Jenner was asked to draw up a plan to prepare an estimate of the cost. The illness of his son necessitated his return to Berkeley, which interrupted this plan, but the warrant for instituting a National Vaccine Establishment was obtained in his absence, and he was appointed Director. Unfortunately, dissension again crept in at the outset, which ended in Jenner's resignation of the post, although he continued to give the institution the benefit of his advice.

In 1810, many domestic trials came upon him. Among these the death of his son distressed him very deeply, and materially affected his health.

The following year he suffered another bereavement in the loss of his sister, which was also a great grief to him. On May 26 of the same year, while he was staying in London, he was summoned to attend the Hon. Robert Grosvenor, who had developed a serious attack of smallpox. He had been vaccinated by Jenner ten years previously. The onset of the disease was very rapid, and in four days he became worse, and serious symptoms manifested themselves. He was attended by Sir Henry Halford, one of the most famous physicians of his time, Sir Walter Farquhar, and Jenner, and, although a fatal termination of the case was regarded as inevitable, he eventually recovered. The publicity given to the matter unfortunately served to revive the agitation against vaccination, and caused quite a panic among those who had had their children vaccinated. A fresh burst of criticism, together with a summons to give evidence before the House of Lords in connection with the Berkeley Peerage, seems to have greatly unnerved Jenner and aged him considerably. In 1814, he visited London for the last time, when he was presented to the Allied sovereigns and the Emperor of Russia, then on a visit to England.

The following year he received another blow, in the loss of his wife, who died after a long illness. Stricken with grief, Jenner retired to Berkeley, which he did not leave again—except for a day or two—until his death on January 26, 1823. He wrote in his last letter to his friend as follows: "I have had an attack from a quarter I did not expect, the 'Edinburgh Review.' These people understand literature better than physick, but it will do incalculable mischief. I put it down at 100,000 deaths at least. Never was I involved in so many perplexities."

His sensitive nature was shaken by the attacks constantly made upon him and his life's work. The day following this letter he retired to rest, apparently in his usual health, and the next morning rose and came down to his library, where he was stricken with an attack of apoplexy and paralysis of the right side while sitting in his favourite chair. He never rallied, and died the following morning, January 26, 1823. He was laid to rest in the family vault in Berkeley Church, Gloucestershire, on February 3, 1823.

The simplicity of Jenner's character and his dislike of ostentation and flattery is shown in his wish recorded by him in a letter with reference to an inscription on any memorial that might be erected to him after his death. He directed it should read:

In Memory of Edward Jenner, LL.D., F.R.C.S., etc.,
who was born May 17, 1749, and died ——
*Not a word more.*

In attempting to estimate Jenner's great achievement, it should be remembered that his discovery did not so much lie in the fact that persons who had been infected with cowpox escaped variolæ, but that the matter taken from a human being suffering from cowpox had the power of protecting another individual from that disease. The lives that his discovery has been instrumental in saving are the most eloquent tribute to his memory, and the principles that he so strenuously advocated and established still remain the only efficient means of protection against one of the most dreadful diseases that afflict mankind.

A unique collection of personal and family relics of Edward Jenner is now exhibited in the Wellcome Historical Medical Museum. They include the original lancets he used in his first experiments, his spectacles, cane, and snuff-box, together with portraits, medals, diplomas, and original MSS.

## Figures and Tables

**Figure f1:**
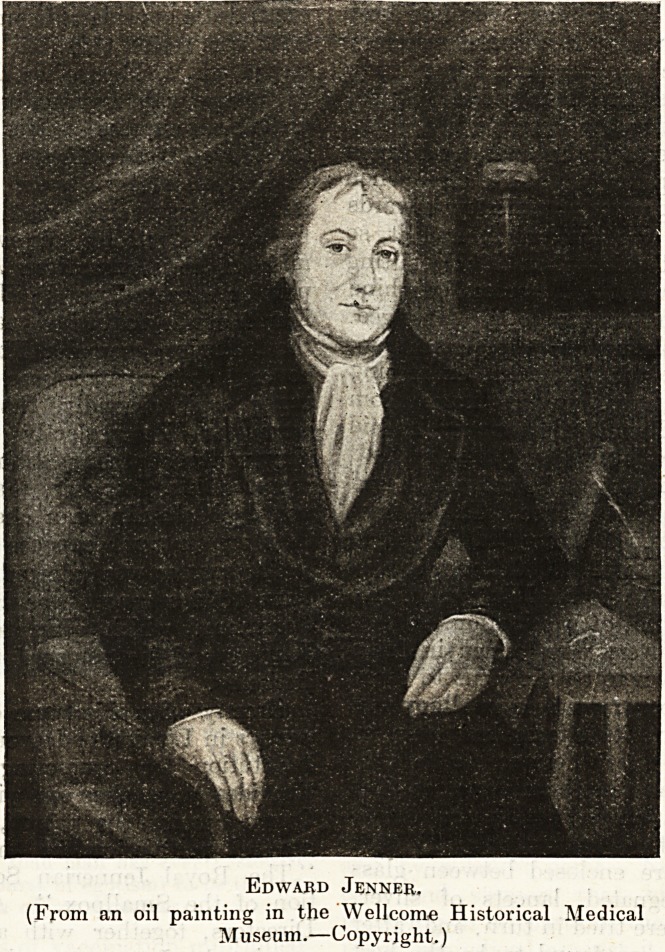


**Figure f2:**